# Myocarditis and Myocardial Diseases—I

**Published:** 1908-08-08

**Authors:** 


(*To be continued.*)

### MYOCARDITIS AND MYOCARDIAL DISEASES—I.

By acute myocarditis is usually meant any febrile condition in which there is evidence of rapid deterioration in the power of the heart's action without mechanical lesion of a valve. By chronic myocarditis is usually meant any condition of heart failure in which, in the absence of renal, pulmonary, or cardiac-valvular lesions, defective power in the musculature of the heart seems to be the sole cause for the failure. The reader may dissent, but these definitions are sufficiently near the mark to form the basis of a useful clinical classification.

The term myocarditis belongs properly to pathology, and if it is used in clinical medicine it should be only for clinical conditions which correspond accurately with the pathological lesion, myocarditis. This, however, is not the case, and seeing that there are no very suitable clinical terms to express the symptom-picture of the various forms of pathological affection of the myocardium, many conditions which give similar clinical symptoms are grouped together as myocarditis. As a matter of fact, when a physician says that, in his opinion, a patient is suffering from acute myocarditis, all that he implies is "one of the acute lesions of the myocardium"; when, on the other hand, he diagnoses chronic myocarditis, he can seldom be sure that it is an inflammation of any kind, for it may be a fibroid, or a fatty-infiltrated heart, or one with obstructed coronary arteries; what he really means is that he thinks his patient has one of the chronic diseases of the myocardium. In clinical medicine it is difficult enough to determine that the pathology of the disease lies in the myocardium at all; it is practically impossible to say whether or not such myocardial affection is inflammatory until one gets to the post-mortem room. Even then microscopical sections must be prepared and examined by a pathologist before the final answer can be given in many cases.

Almost all the acute affections of the myocardium afford the same clinical picture; almost all the chronic affections of the myocardium, if they are of clinical importance at all, afford another clinical picture. From the bedside point of view, therefore, myocardial affections may be divided into acute and chronic. It is not proposed to deal with the symptoms of these here, for they are well enough known; but having made it clear that the pathological lesions of the myocardium by no means coincide with the clinical groups, we shall leave the latter for the present and devote attention entirely to the former.

The morbid conditions which will be shortly discussed in turn are the following:—

A. *Acute Lesions of the Myocardium.*—(i.) Acute cloudy swelling; (ii.) acute segmentary degeneration; (iii.) myomalacia cordis; (iv.) acute general interstitial inflammation; (v.) acute focal interstitial inflammation.

B. *Chronic Lesions of the Myocardium.*—(i.) Fatty overgrowth, or superposition; (ii.) fatty infiltration; (iii.) fatty degeneration; (iv.) general interstitial fibrosis—fibroid heart; (v.) local fibrosis; (vi.) brown pigmentary degeneration.

Before proceeding, and without recurring to the errors of the use of the term myocarditis in clinical medicine, it may be of some interest to discuss which of the above myocardial affections merit the term myocarditis from the strictly pathological point of view. Even here there will be difference of opinion. There are only two of the lesions about which there can be little or no question, namely, acute general interstitial inflammation and acute focal (pyæmic) interstitial inflammation. Each of these will be allowed the term "acute interstitial myocarditis" by all. When we come to lesions of the parenchyma, however, with little or no affection of the interstitial tissue, are we to call acute cloudy swelling an acute myocarditis or not? Similarly in regard to acute segmentary degeneration and myomalacia cordis. Some would term these all inflammatory; some would call the first two inflammatory, but would exclude myomalacia cordis as being a necrotic degeneration. Others, again, would rather use the terms acute cloudy swelling and so forth, for clearness sake, rather than quarrel as to whether the changes are really inflammatory or not. Thus these authorities would restrict the term myocarditis to those cardiac conditions in which there is acute interstitial mischief.

The same applies to the chronic lesions. Some would term an ordinary fibroid heart a chronic myocarditis; others deny this, and point out that fibrosis is not itself an inflammation, though it may be the result of—the repair of—a former inflammation which has ceased. It seems best, therefore, not to use the term chronic myocarditis at all, but to specify the actual change in the myocardium rather than the process by which it may be thought to have come about. Confusion will thus be avoided from the outset.

